# Advances in the Microbiome: Applications to *Clostridium difficile* Infection

**DOI:** 10.3390/jcm5090083

**Published:** 2016-09-21

**Authors:** Eamonn P. Culligan, Roy D. Sleator

**Affiliations:** Department of Biological Sciences, Cork Institute of Technology, Bishopstown, Cork, Ireland; eamonn.culligan@cit.ie

**Keywords:** *Clostridium difficile*, bacteriocin, human gut microbiome, gut microbiota, antibiotic resistance, faecal microbiota transplantation (FMT), probiotics, genome sequencing, nanopore sequencing, synthetic biology

## Abstract

*Clostridium difficile* is a major cause of morbidity and mortality worldwide, causing over 400,000 infections and approximately 29,000 deaths in the United States alone each year. *C. difficile* is the most common cause of nosocomial diarrhoea in the developed world, and, in recent years, the emergence of hyper-virulent (mainly ribotypes 027 and 078, sometimes characterised by increased toxin production), epidemic strains and an increase in the number of community-acquired infections has caused further concern. Antibiotic therapy with metronidazole, vancomycin or fidaxomicin is the primary treatment for *C. difficile* infection (CDI). However, CDI is unique, in that, antibiotic use is also a major risk factor for acquiring CDI or recurrent CDI due to disruption of the normal gut microbiota. Therefore, there is an urgent need for alternative, non-antibiotic therapeutics to treat or prevent CDI. Here, we review a number of such potential treatments which have emerged from advances in the field of microbiome research.

## 1. Introduction

The human gastrointestinal (GI) tract harbours hundreds of microbial species (gut microbiota) and thousands of strains, and is one of the most densely populated ecosystems on Earth [1]. The oft-stated claim that bacterial cells in the gut outnumber human cells in the body by a ratio of 10:1 has recently been revised and proposed to be approximately a 1:1 ratio [2]. Nevertheless, the actual ratio and number of cells is not as important as the functional capacity of the gut microbiota, which has numerous positive benefits for host health and physiology; including enhanced energy harvest, vitamin synthesis, modulating intestinal and immune cell proliferation and development, and providing protection against infection [3,4,5,6,7,8]. Disruption of the “normal” gut microbiota composition has important implications for human health and disease, having been linked to conditions such as inflammatory bowel disease (IBD), irritable bowel syndrome (IBS), cancer and obesity [9,10,11]. In many cases, more evidence is needed to definitively link the microbiota and disease, and it is unknown if an altered microbiota is a cause or effect of the disease state [12]. In the case of *Clostridium difficile* infection (CDI), the majority of cases are undoubtedly linked to altered gut microbiota composition, usually following administration of antibiotics to the host [13]. Here, we review how advances in microbiome research have revealed novel opportunities for the control of *C. difficile*.

## 2. *Clostridium difficile*

*C. difficile* is a Gram-positive, anaerobic, spore-forming bacterium. Once established in the gut, *C. difficile* produces two main toxins, Toxin A (TcdA) and Toxin B (TcdB), which cause enterotoxic, cytotoxic and inflammatory damage to intestinal cells [14]. TcdA and TcdB are members of the large clostridial glucosylating toxin (LCGT) family. Both toxins target cellular GTPases of the Rho and Ras families [15]. TcdA was initially proposed as the main virulence factor in *C. difficile* [16]. More recently, it was reported that only TcdB is essential for virulence [17]. A later, conflicting study showed that both TcdA and TcdB are important for *C. difficile* infection [18,19], although TcdA-negative strains of *C. difficile* have been identified in clinical isolates [20,21]. The two major toxins, TcdA and TcdB, are encoded by the *tcdA* and *tcdB* genes, respectively, which are located on the “PaLoc” pathogenicity locus. Some strains of *C. difficile* may also encode the *C. difficile* transferase (CDT) binary toxin. Non-toxigenic strains of *C. difficile* are not usually associated with human disease, but it has been shown that horizontal transfer of PaLoc can allow non-toxigenic *C. difficile* to produce active toxins [22].

*C. difficile* is one of the most notorious gastrointestinal pathogens and is the most common identifiable cause of infectious nosocomial diarrhoea in developed countries and the major cause of gastroenteritis in nursing homes and health care facilities for the elderly [23,24]. In recent years, increases in the frequency and severity of CDI have been observed, as well as increased risk of community-associated CDI and CDI in persons previously thought to be low risk [25,26,27]. It is estimated that CDI affects up to 1.2% of hospitalized patients in the United States, representing an estimated cost of $433–$797 million per year [28,29,30]. In Europe, the estimated cost is approximately €3 billion per year, which is likely to increase concomitant with a more elderly society; more than 134 million Europeans will be >65 years by 2050 [31].

**Figure 1:**
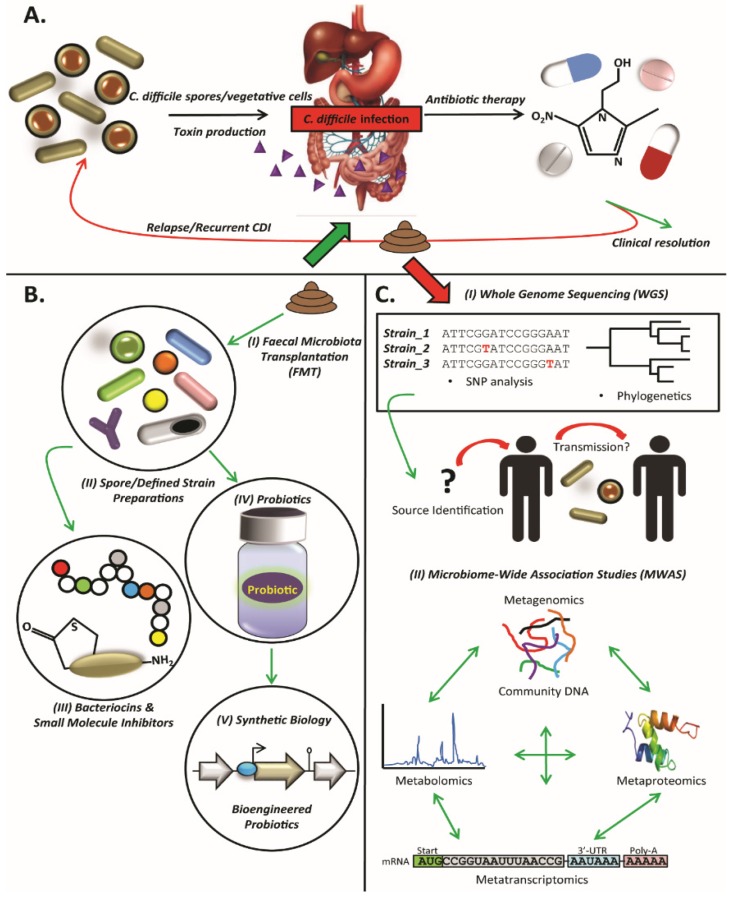
(**A**) Overview of *C. difficile* infection (CDI). CDI results from ingestion of *C. difficile* spores, which germinate to vegetative cells in the gastrointestinal tract. *C. difficile* produces potent toxins, which cause enterotoxic, cytotoxic and inflammatory damage to intestinal epithelial cells. Primary treatment of CDI with antibiotics (e.g., metronidazole, vancomycin or fidaxomicin) can lead to clinical resolution of infection, but in some cases, relapse can occur causing recurrent CDI; (**B**) Overview of potential non-antibiotic therapeutic alternatives for CDI. (I) Faecal microbiota transplantation (FMT) involves the transfer of a whole stool preparation from a healthy donor to a patient with CDI. Cure rates of ~90% have been reported from numerous trials of patients with CDI, making FMT one of the most promising non-antibiotic therapeutics; (II) Use of defined mixtures of bacterial strains or spore preparations have also been trialled for treatment of CDI; (III) Antimicrobial compounds produced by bacteria, such as bacteriocins and small molecule metabolites have been identified, some with potent anti-*C. difficile* activity (e.g., thuricin CD); (IV) Probiotic strains of bacteria and yeast may hold some promise for the prevention of CDI, when administered as adjunct therapies with antibiotics; (V) Integrating developments in synthetic biology and genetic engineering may enable the development of bioengineered probiotics, which target specific pathogens and toxins; (**C**) (I) Whole genome sequencing (WGS) has the potential to revolutionise pathogen diagnostics. Ultra-fine resolution single nucleotide polymorphism (SNP) analysis and phylogenetic reconstructions can help improve the identification of the source of an infectious disease outbreak, as well as potential transmission events. Improvements to DNA sequencing technologies, such as Oxford Nanopore’s MinION, could also dramatically reduce the time from sample isolation to pathogen identification; (II) Similarly, microbiome-wide association studies (MWAS) have the potential to improve our understanding of disease dynamics and the complex interactions between pathogen and the host microbiota during infection, by integrating approaches such as metagenomics, metaproteomics, metatranscriptomics and metabolomics.

In addition to hospitalization, the most significant predisposing factors for CDI include advanced age (>65 years) and antibiotic therapy (disrupts the normal gut microbiota) [32]. The most common antibiotics implicated to date include broad-spectrum cephalosporins, fluoroquinolones and clindamycin [33,34,35]. The only remaining effective therapeutic agents are metronidazole, vancomycin and fidaxomicin [36,37]. Against this backdrop, the last decade has seen the emergence of a new epidemic of CDI [38] characterised by increased frequency and severity of enteric disease and increased resistance to antibiotic therapy. Faced with this epidemic, clinicians are struggling to find viable therapeutic alternatives [37]. An overview of the cycle of CDI is presented in Figure 1A.

The following sections outline a number of alternative, non-antibiotic approaches that have potential to control *C. difficile*. 

## 3. Faecal Microbiota Transplantation 

Faecal microbiota transplantation (FMT) is the transfer of a suspension of faecal material from a healthy donor to a recipient with aim of re-establishing a “normal” microbiota profile, usually for the treatment of recurrent CDI. Delivery of faecal material to recipients is usually either via rectal enema, naso-duodenal tube or colonoscopy. Recommendations state that if there are three or more recurrences of CDI following pulsed vancomycin therapy, FMT should be considered the next therapeutic option [39]. FMT therapy for CDI has repeatedly provided promising results for treatment of CDI. In a landmark randomised, open label, clinical trial, van Nood and colleagues, compared FMT, vancomycin and bowel lavage to vancomycin and bowel lavage and vancomycin alone [40]. Overall, a 94% cure rate (15/16 patients) was observed for the FMT group, with 81% (13/16 patients) cured after one FMT dose. Two further patients were cured following a second FMT. This compared to 23% and 31% cure rates in the vancomycin-bowel lavage and vancomycin alone groups, respectively. The trial was suspended early, as it was deemed unethical not to provide FMT treatment to the control groups. Furthermore, increased microbiota diversity was noted in FMT recipients, with increases in Bacteroidetes, *Clostridium* clusters IV and XIVa, and decreases in Proteobacteria [40]. Another recent clinical trial, reported a 90% cure rate for FMT compared to 23% for vancomycin-treated control group [41]. Such high cure rates are not uncommon for FMT treatment for CDI. A systematic review of FMT reported a mean cure rate of ~91% [42] and a similar study with a meta-analysis assessing long-term (>3 months) efficacy and safety of 18 different studies including >600 patients also reported a primary cure rate of ~91% [43]. A number of additional studies have also reported similar cure rates [44,45]. 

Despite the success rates, general efficacy and low incidence of adverse events associated with FMT, there are legitimate safety and regulatory concerns. While most adverse events are mild to moderate (e.g., bloating, flatulence, abdominal discomfort and diarrhoea), long-term side effects which are currently unpredictable or unknown, may emerge in the future. Therefore, larger longitudinal trials, which monitor patients over a period of years rather than months, are required. Brandt et al., reported four patients with new conditions post-FMT, including peripheral neuropathy, Sjorgren’s syndrome, idiopathic thrombocytopenic purpura and rheumatoid arthritis, but it is unknown if these are related to FMT [44]. Another study reported microscopic colitis, Sjorgren’s syndrome, follicular lymphoma, contact dermatitis, idiopathic Bence-Jones proteinuria and laryngeal carcinoma post-FMT, but these were thought to be associated with unrelated, pre-disposing factors [46]. Additional rare adverse events, such as bacteraemia, fever, colonic perforation and death, are reviewed by Baxter and Colville [47]. Even with rigorous screening of donor faecal samples for any potential major pathogens [48] it is not possible to screen for all. Furthermore, FMT is sometimes erroneously referred to as faecal bacteriotherapy, which is plainly wrong, as faeces is a complex mix of bacteria, as well as archaea, fungi, viruses, human cells, metabolites and more [49]. Despite the risks, many of which are currently unknown, the exceptionally high cure rate and the fact that FMT may be a last resort treatment following repeated failure of antibiotic therapy make FMT a life-saving option for the treatment of CDI in many cases.

Regulation of FMT has also become an important issue. The Food and Drug Administration (FDA) in the United States had initially classed FMT as a drug and biological product and, as such, brought FMT under the same remit and regulations as traditional pharmaceutical drugs. This decision was soon revised to exercise discretion in cases where FMT was to be used in the case of CDI [50]. As of yet, FMT has not been regulated in Europe, as far as we are aware. Regulation of FMT is complicated by the multifarious nature of faecal samples. No two samples from different individual donors will be the same, so how does one apply a regulatory framework to what are essentially different “products”? The answer is not immediately obvious and there are concerns that over-regulation or granting of exclusivity to one stool-bank supplier may increase the cost of FMT and drive desperate patients to the precarious area of “do-it-yourself” FMT [50]. Other authors have argued that FMT could be regulated as tissue, rather than a drug or given its own classification altogether [51]. Obviously some regulation is required, the extent of which may lie somewhere in the middle of no regulation and regulation as a drug. Given the unique nature of the procedure and the transplanted faecal material, a stand-alone classification may be the most practical solution.

## 4. Defined Strain or Spore Formulations

A similar approach to FMT, but using a more defined microbiota mixture, a number of studies have investigated using single or a defined number of strains to compete with *C. difficile*. Khanna et al., assessed the efficacy of a spore mixture (SER-109) to prevent recurrent CDI in a phase Ib clinical trial [52]. Stool samples from healthy donors were screened, processed and treated with ethanol to kill vegetative cells. Overall, SER-109 was administered to 30 patients; 26 (86.7%) of which had no recurrent *C. difficile* associated diarrhoea up to the eight-week end-point of the study. SER-109 was well tolerated, but some mild to moderate adverse events were noted, such as diarrhoea, nausea and abdominal pain. Furthermore, gut microbiota diversity was increased following treatment, including commensal species not present in the SER-109 spore preparation. Although the study lacked a placebo group, the results were nevertheless promising and demonstrated a potentially safer method than FMT, where whole faecal preparations are administered, rather than purified spores [52]. Despite the initially promising results of the phase Ib trial, a subsequent phase II trial of SER-109 failed to achieve the primary efficacy endpoint of reduced CDI occurrence after 8 weeks. A recent press release from the company, Seres Therapeutics, detailing the interim results of the trial can be found at the following link: http://ir.serestherapeutics.com/phoenix.zhtml?c=254006&p=RssLanding&cat=news&id=2190006. 

The failed SER-109 trial was surprising to the company and observers alike, but perhaps serves as a reminder that the gut microbiome is an exquisitely complex ecosystem and may not be as easily manipulated as we would hope.

A spore preparation of non-toxigenic *C. difficile* (NTCD) has also been assessed to prevent recurrent CDI. The phase II, randomised, double-blind, placebo-controlled trial included 168 patients. The trial drug was concluded to be well tolerated and safe, with relatively few adverse events noted. CDI recurrence was reported in 11% of patients receiving spore treatment and in 30% of placebo controls. Interestingly, successful colonisation with spore treatment was associated with lower recurrence, indicating additional factors may influence infection resistance; 2% of patients who were colonised with NTCD spores suffered recurrence, compared to 31% in non-colonised patients [53]. One serious area of concern with using NTCD is that horizontal transfer, albeit at low frequency, of the PaLoc (containing the genes encoding TcdA and TcdB) has been demonstrated experimentally [22] and also shown to have occurred in circulating *C. difficile* populations [54]. Of further concern, is the co-transfer of conjugative transposons encoding antibiotic resistance genes with PaLoc [22]. Clearly, creating a situation where NTCD can become toxigenic is highly undesirable; the risks and the conditions that favour the transfer of PaLoc will need to be determined prior to widespread adoption of NTCD spores as a treatment. This point is further reinforced by the isolation of toxin-negative *C. difficile* strains from humans and animals with GI disease symptoms [55]. The authors state that disease symptoms were likely due to non-toxigenic *C. difficile,* but they could also have been due to an unknown or uncultured organism. Nevertheless, knowledge of the pathogenicity and virulence of non-toxigenic strains is limited and quite ambiguous, requiring further research.

Another recent study identified 11 bacterial operational taxonomic units (OTUs) associated with resistance to *C. difficile* infection [56]. The OTUs originated mainly from *Clostridium* cluster XIVa, while *Clostridium scindens* was the species most strongly associated with infection resistance. In addition, using a systems biology approach and mathematical modelling, *C. scindens* was also identified from human models. Culturing and transfer of a mixture of the four OTUs most strongly associated with resistance, or transfer of *C. scindens* alone to mice reduced *C. difficile* infection and mortality. Furthermore, the authors unravelled a potential mechanism of action for *C. scindens* inhibition of *C. difficile* through secondary bile acid modification. *C. scindens* possesses the *baiCD* (bile acid inducible) locus, which encodes 7a-hydroxysteroid dehydrogenase, an essential enzyme for dihydroxylation and secondary bile acid biosynthesis, of which, deoxycholate and lithocholate inhibit *C. difficile*. 

In a proof on concept study, representative species from a healthy donor stool sample were cultured and combined as a stool substitute (“RePOOPulate”). Thirty-three species were included in the stool substitute following culturing and administered to two patients who had not responded to antibiotic treatment for recurrent CDI (hyper-virulent *C. difficile* 078). Both patients formed normal stool within two to three days of treatment and did not relapse despite receiving antibiotics for additional, unrelated infections. Both patients remained free of recurrent CDI after six months follow-up [57]. A similar study has previously been shown to be successful for the prevention of recurrent CDI in mice, where the authors used a defined, six-species cocktail of bacteria including *Staphylococcus warneri*, *Enterococcus hirae*, *Lactobacillus reuteri*, and novel species from the genera *Anaerostipes*, *Bacteroides* and *Enterorhabdus* [58].

Overall, these studies represent promising results for the use of defined bacterial formulations for the treatment of CDI. Larger studies are needed however, and it is unlikely there is a “magic-bullet” mixture of species or strains that will work for everyone. Complex interactions and differences in the composition of the gut microbiota of different donors and recipients, host immune factors and genetics are all likely to influence the success or otherwise of such treatments.

## 5. Microbiome-Wide Association Studies (MWAS) 

Microbiome-wide association studies (MWAS) integrate systems biology approaches such as metagenomics, metatranscriptomics, metaproteomics and metabolomics with the aim of identifying complex interactions between microbial communities and potential links to health and disease [59]. For example, by combining metagenomics and metabolomics with animal infection studies, Koenigsknecht and colleagues determined the dynamics of CDI in mice [60]. *C. difficile* colony counts increased from 10^2^ CFU/g of intestinal contents to 10^8^ CFU/g by 18 h post-infection in the distal GI tract and were found throughout the entire GI tract at 36 h (time of death). Toxins were detected at 24 h post challenge, which overlapped with the first detection of spores and the number of spores was found to be highest in the large intestine. In addition, the first evidence of histopathological damage was seen at 30 h, which coincided with the first outwardly observable signs of illness in the mice. Metagenomic sequencing revealed changes to the microbiota over the time-course of the experiment: members of the *Lactobacillaceae* eventually dominated the distal small intestine and *C. difficile* dominated the large intestine. Furthermore, metabolomic analysis revealed that antibiotic treatment altered the composition of bile acids in the intestine, most notably, the depletion of secondary bile acids (which can inhibit vegetative *C. difficile*) [60]. Antibiotic treatment likely destroys members of the commensal microbiota that metabolise primary bile acids to secondary bile acids, leaving a greater pool of primary bile acids which promote *C. difficile* spore germination. Another recent integrative study has also shown that levels of primary bile acids were significantly higher in stool samples from patients with recurrent CDI compared to controls and certain bile acid ratios could be used to correctly differentiate between patients with a first episode of CDI and patients with recurrent CDI ~84% of the time [61]. The *C. scindens* study [56] outlined above is also a good example of a MWAS. Integrating metagenomic and 16S rRNA sequencing, animal models, clinical data and mathematical modelling identified *C. scindens* as a potential species which aids resistance against CDI, which was proved by transplanting *C. scindens* to mice receiving antibiotics and challenged with *C. difficile* [56]. These studies illustrate how the power of a combined approach to studying the microbiome can lead to definitive links between the microbiome and diseases such as CDI. Future work will focus on the challenge of translating this knowledge into clinically relevant diagnostics, prognostics and treatments. MWAS could also be used to track combined changes in microbiota composition, gene expression and metabolomic profiles over time following FMT to treat CDI [59]. While still in its infancy, a MWAS approach could reveal novel insights regarding CDI in the future.

## 6. Whole-Genome Sequencing

Improvements to DNA sequencing technologies, reduced costs and increased throughput is bringing whole-genome sequencing (WGS) to the forefront of clinical microbiology, pathogen diagnostics and epidemiology. For example, Public Health England (PHE) are now characterising all potential *Salmonella enterica* isolates using WGS [62]. Similarly, the Centres for Disease Control (CDC) and collaborators have implemented the “*Listeria* Whole-Genome Sequencing Project” for all *Listeria* isolates in the United States [63]. WGS provides higher-resolution data (WGS can detect insertion, deletion and recombination events in genome sequences, as well as single nucleotide polymorphisms (SNPs)) compared to typing methods such as pulse-field gel electrophoresis (PFGE), multi-locus sequence typing (MLST) and serotyping, enabling improved identification of an outbreak source and potential transmission events [64]. Numerous studies have demonstrated the effectiveness of WGS for analysing and tracking outbreaks of clinically relevant pathogens, such as *Staphylococcus aureus*, *Acinetobacter baumannii*, *Klebsiella pneumoniae* and *Pseudomonas aeruginosa* [65,66,67,68]. 

A number of studies have also focused on *C. difficile*. WGS and phylogenetic analysis were used to track transmission of epidemic clones of *C. difficile* 027 among patients in hospital wards over a two-year period [69]. High-resolution SNP analysis following WGS identified 27 distinct genotypes and 32 potential occasions transmission occurred either directly or indirectly. In addition, highly contagious donors, who infected numerous other patients, were also identified. Recurrent infections, either by the same or by a different strain could be discriminated even up to 26 weeks duration. Similarly, Mac Aogain and colleagues found recurrent CDI outside the stated eight-week cut-off [70]. Additionally, WGS distinguished between relapse (with the same strain of *C. difficile*) and reinfection (with a different strain), and could identify within-strain evolution by characterising emerging SNPs during the course of recurrent infection. A number of SNPs caused non-synonymous mutations in protein-coding genes, some of which encode virulence regulators and therefore have important clinical implications [70]. Jia et al., identified two outbreaks in a hospital in mainland China and used WGS to reconstruct a transmission map and trace the source of a bloodstream infection to a *C. difficile* 027 strain rarely found in China [71]. 

Outside of a hospital ward environment, WGS has also been used to track and distinguish *C. difficile* isolates on a national scale. For example, Eyre et al. investigated an Australia-wide outbreak of an uncommon *C. difficile* 244 ribotype. An unusually high proportion of infections appeared to be community-acquired and in patients <65 years old. Although the source could not be identified, isolates were very closely related genetically, even though they were isolated from locations all across Australia and separated by thousands of kilometres. The authors consider an animal or food as a potential source. WGS also revealed spread of the outbreak strain to Southampton, England, by an individual who had travelled from Australia during this time [72]. WGS has also been used to map the introduction and spread of fluoroquinolone-resistant *C. difficile* 027 in Germany [73]. Furthermore, WGS has identified identical and near identical (<2 SNPs between isolates) strains of *C. difficile* in both farmers and pigs on farms in the Netherlands, indicating potential transmission between both, while some strains also shared mobile tetracycline and streptomycin resistance determinants [74].

The emergence of novel sequencing technologies, such as Oxford Nanopore’s MinION sequencer (www.nanoporetech.com), could revolutionise pathogen diagnostics and treatment in the field. This portable DNA sequencer can be connected to any suitable laptop or PC and provide rapid, long read, real-time DNA sequencing. While the platform is still optimising and improving accuracy and error rates, a number of studies have already demonstrated its potential. Retrospective sequencing of samples from a *Salmonella* outbreak, which had been sequenced previously using an Illumina MiSeq, were sequenced using the MinION. It was possible to identify the species as *S. enterica* 30 min after loading the sample, identify the likely serovar as Enteritidis after 40 min and confidently assign the strain to the outbreak cluster within 100 min [75]. This technology has since been used for real-time genomic surveillance of the recent Ebola epidemic in West Africa [76]. Furthermore, a recent study has developed a method to selectively sequence only specific regions of interest in DNA. The method termed “Read Until” uses a predetermined squiggle pattern from the sequence of DNA of interest as a reference and rejects non-matches, only sequencing regions that match the reference squiggle [77]. Further development and optimisation of this approach could enable extremely rapid pathogen diagnostics.

Currently routine WGS in clinical microbiology is most suited to public health and reference laboratories and is unlikely to be adopted on a widespread basis by individual hospitals in the short-term and many factors need to be considered, including cost, infrastructure, sequencing capacity and data analysis capabilities [78]. Further cost reductions, technological developments and perhaps most importantly, overcoming challenges associated with bioinformatic analysis [78,79,80] will all aid the more widespread adoption of WGS in clinical settings. Finally, a recent study has shown that significant monetary savings (in excess of €200,000 in this case) can be achieved by adopting WGS to inform more targeted infection control policies [81]. If such savings can be consistently demonstrated, they are likely to be key in deciding whether to adopt routine WGS and would also offset a proportion of the setup costs.

## 7. Probiotics

Probiotics, defined as “products that deliver live micro-organisms with a suitable viable count of well-defined strains, with a reasonable expectation of delivering benefits for the wellbeing of the host” [82], have been investigated for many years as a therapy to prevent CDI and other gastrointestinal conditions [83,84]. 

One observational study assessed the efficacy of a three-strain probiotic mixture, BioK+^®^ (containing *Lactobacillus acidophilus* CL1285, *Lactobacillus casei* LBC80R and *Lactobacillus rhamnosus* CLR2), on all adult patients given any antibiotic, to prevent CDI. The probiotic was given within 2–12 h following antibiotic administration and continued for at least 30 days or until the course of antibiotic was finished. A 39% reduction in the rate of CDI cases was seen and this intervention was monitored over 10 years. Overall, almost 45,000 patients received the probiotic and the CDI rate decreased from 18.0 cases per 10,000 patient-days to an average of 2.3 cases per 10,000 patient-days, in which time the incidence of CDI remained low [85]. An earlier study using the same probiotic preparation (BioK+^®^) for prevention of antibiotic-associated diarrhoea (AAD) and CDI, compared three treatment groups (two probiotic capsules per day (Pro-2), one probiotic and one placebo capsule per day (Pro-1) and two placebo capsules per day). Both probiotic groups had a lower incidence of AAD and shorter symptom duration. Furthermore, the incidence of CDI was lower for both probiotic groups (1.2% and 9.4% for Pro-2 and Pro-1 groups, respectively) compared to 23.8% for the placebo group [86]. A number of systematic reviews and meta-analyses have also determined that reduced rates of CDI are associated with providing probiotics as an adjunctive treatment to patients receiving antibiotics [87,88,89].

The efficacy of a species of yeast, *Saccharomyces boulardii*, has also been tested in a number of clinical trials. *S. boulardii* treated patients were found to have significantly lower rates of CDI recurrence (26%) compared to placebo-treated groups (45%) in a phase III clinical trial [90]. Another phase III trial compared *S. boulardii* in combination with high and low dose vancomycin or metronidazole versus placebo. Only *S. boulardii* and high-dose vancomycin therapy showed a lower CDI recurrence rate (17%) compared to the placebo group (50%) [91]. A further trial however, found that *S. boulardii* was not effective at preventing AAD in elderly patients [92]. 

Two randomised, controlled trials using *Lactobacillus rhamnosus GG* and *Lactobacillus plantarum* 299v did not demonstrate efficacy of probiotic treatment over placebo for the prevention of AAD, although it is difficult to draw definitive conclusions due to the small sample size of study participants [93,94]. A multi-centre, randomised, double-blind, placebo-controlled trial found no benefit to probiotic (mixture of two *Lactobacillus* and two *Bifidobacterium* strains) administration in the prevention of CDI in more 2941 patients >65 years of age [95,96].

Overall, there appears to be evidence supporting the theory that adjunctive probiotics can help prevent CDI in some cases (mainly primary CDI, rather than secondary prevention of recurrent CDI [97]). There is a need for much larger multi-centre, randomised, double-blind, placebo controlled trials as the apparent strain-specific efficacy, single or multiple strain combinations and other confounding factors such as composition of patients’ gut microbiota, host immune status and genetics, all likely contribute to success or failure of different probiotic strains.

An alternative approach, which has been gaining significant traction in recent years, is the application of synthetic biology and bioengineering of microorganisms to target specific pathogens [98,99]. Significant research has focused on improving the robustness of certain probiotic strains to enhance survival during passage through GI tract and ultimately increase persistence and the timeframe to elicit a therapeutic benefit [100,101,102,103,104,105]. Additionally, probiotics have been genetically modified to kill specific pathogens. *Escherichia coli* was engineered to detect and move toward *P. aeruginosa* by modifying a chemotaxis protein (CheY) and subsequently produce an anti-*Pseudomonas* bacteriocin and nuclease to destroy both planktonic cells and biofilms in response to a trigger molecule produced by *P. aeruginosa* (*N*-acyl homoserine lactone; AHL) [106]. A similar bacteriocin-mediated killing approach was developed also to target *P. aeruginosa* following lysis of the producing strain [107]. Bioengineered probiotics have also been developed which target and bind bacterial toxins. By expressing genes, which encode oligosaccharide moieties, probiotics display modified cell surface proteins, which mimic core structures of host cell receptors for various toxins. For example, Paton and co-workers developed an *E. coli* strain expressing a receptor mimic, which could bind Shiga toxin (Stx) and protect mice from a fatal dose of Shiga-toxigenic *E. coli* (STEC) [108]. Similarly, bacteria engineered to bind enterotoxigenic *E. coli* (ETEC) labile toxin and cholera toxin (Ctx) have also been successfully developed [109,110].

More recently, a novel system has been developed in *Salmonella.* A genetic circuit was created to induce cellular lysis at a certain level of population density in response to AHL. Upon lysis, the engineered strain releases a haemolytic toxin, encoded by the *E. coli hlyE* gene. A low number of bacteria survive and repopulate the environment and can begin the cycle again, creating synchronised, oscillatory cycles of toxin/drug delivery. Furthermore, the engineered strain was demonstrated to decrease tumour activity and increase mean survival time by approximately 50% in mice [111]. While not a trivial undertaking, such a system could be adapted to incorporate a different delivery strain, regulatory control mechanism, drug/toxin or target organism or cell type. We are not aware of any bioengineered probiotics that have as yet, been developed to target *C. difficile*, but it may prove an interesting area to pursue in the future, by tailoring or combining some of the examples above to specifically target *C. difficile* as outlined by Sleator and Hill [112].

## 8. Small Molecule Inhibitors

The mammalian GI tract contains thousands of small molecule compounds, many of which are uncharacterised [113]. Such molecules play roles in intra- and intercellular communication (e.g., host-microbe, host-host and microbe-microbe) and may inhibit cellular processes. For example, Antunes et al., identified small molecules from human faecal sample extracts that could repress expression of invasion genes in *Salmonella* [114]. Most notably, repression of genes on *Salmonella* Pathogenicity Island 1 (SPI-1) and their global regulator, HilA, were among the most repressed and in vitro cell culture invasion was reduced 96% in the presence of the faecal extracts. Furthermore, production of the small molecule inhibitor was assigned to *Clostridium citroniae* and closely related strains from *Clostridium* cluster XIVa [114]. While the above example does not target *C. difficile*, it does highlight the potential to identify anti-virulence molecules from within the gut microbiome. A similar screening approach targeting *C. difficile* may identify novel small molecules with therapeutic potential. 

Such inhibitors could also be important following the recent discovery that synthesis of *C. difficile* TcdA and TcdB is regulated by a novel quorum-signalling thiolactone molecule (which could be detected in the stool of patients with CDI) [115]. This is an important finding as, as we have mentioned previously, non-toxigenic strains are not usually associated with disease. Therefore, identifying an inhibitor of the thiolactone inducer represents a potentially exciting target for non-antibiotic *C. difficile* inhibition. Work to characterise the structure of the thiolactone is ongoing and once identified, screening for inhibitors from the gut microbiome or chemical compound banks could begin.

A number of anti-*C. difficile* small molecules have been identified with varying levels of efficacy. An anti-virulence compound which targets TcdB has been described [116]. The compound, Ebselen (2-phenyl-1,2-benzoselenazol-3-one), was identified from a high-throughput screen of a library of bio-available drugs and functions by inhibiting glucosylation of Rho and Rac GTPases by preventing auto-processing of TcdB. The compound also improved survivability and reduced disease symptoms in a murine model of CDI. Perhaps most promisingly, Ebselen has been used in phase II clinical trials for unrelated conditions and has been found to be safe and well tolerated in humans, which may expedite its use for CDI [117]. Similarly, Tam et al. identified a number of compounds which target TcdB. Interestingly, one of the compounds was a bile acid derivative, methyl cholate, which prevented TcdB binding to cell receptors [118]. 

Knowledge from such studies could also inform microbiome screening for similar molecules. For example, biological therapeutic molecules produced by probiotic microorganisms may also provide novel compounds to target *C. difficile*. Ripert and co-workers identified a protease (encoded by the *aprE* gene), produced by the probiotic strain *Bacillus clausii* O/C, which could protect Vero and Caco-2 cell lines from the cytotoxic effects of *C. difficile* [119]. Furthermore, *B. clausii* culture supernatant prevented *C. difficile*-toxin-mediated upregulation of RhoB, which contributes to the cytotoxic effects and apoptosis [120].

## 9. Bacteriocins

Bacteriocins are a diverse family of ribosomally synthesised, antimicrobial peptides produced by bacteria. Hundreds of bacteriocins have been characterised which can have broad- or narrow-spectrum activity against a range of Gram-positive and Gram-negative bacteria (see Cotter et al. for an extensive review [121]). Thousands more bacteriocins and other antimicrobials remain uncharacterised, but have been identified in genome and metagenome sequence datasets and therefore represent a large pool of potentially novel antimicrobials [122,123,124]. 

As mentioned previously, the human microbiome collectively encodes thousands of antimicrobial compounds. A recent study identified a novel peptide antibiotic, lugdunin (produced by a nasal isolate of *Staphylococcus lugdunensis*), with in vitro and in vivo activity against a number of pathogens, including methicillin-resistant *S. aureus* (MRSA) [125]. Another study showed *Enterococcus faecalis* expressing a plasmid-encoded bacteriocin could displace vancomycin-resistant enterococci (VRE); indicating bacteriocin production can provide a competitive advantage and influence niche colonisation [126]. In the case of *C. difficile*, microbiome screening identified a novel two-component bacteriocin produced by *Bacillus thuringiensis* [127]. Approximately 30,000 isolates from a human faecal sample were screened for activity against *C. difficile*. One isolate produced a potent, narrow-spectrum bacteriocin (thuricin CD), active in the nanomolar range. Perhaps the most attractive feature of thuricin CD, is that it displays negligible activity against the commensal gut microbiota, making it an extremely attractive therapeutic candidate. Further work revealed that thuricin CD is as effective as metronidazole and vancomycin against *C. difficile* in a distal colon model [128]; however, degradation and availability in the gastrointestinal tract may be an issue to be overcome if thuricin CD is to be developed as a therapeutic [129]. Interestingly, similar gene clusters to that which encode thuricin CD have been identified in genome and metagenome datasets using a bioinformatics approach, indicating that further novel variants with therapeutic potential are yet to be characterised [130].

Nisin, the archetypal bacteriocin, is produced by many strains of *Lactococcus lactis* and has been used for many years as a food additive. Bartoloni et al., assessed the in vitro efficacy of nisin against 60 clinical isolates of *C. difficile* compared to vancomycin and metronidazole. Nisin was found to be the most active of the three compounds and had the lowest minimum inhibitory concentration (MIC) range [131]. Nisin has also been shown to be effective at killing *C. difficile* in a model human colon, albeit at high concentrations (20 times the MIC; 76 µmol/L). However, the higher concentration was also associated with undesirable alterations to the commensal microbiota, with reductions in *Ruminococcaceae*, *Lachnospiraceae*, *Lactobacillaceae/Leuconostocaceae* and bifidobacteria [132]. 

Lacticin 3147 is another bacteriocin produced by strains of *L. lactis* [133] with potent anti-*C. difficile* activity. Lacticin concentrations as low as 18 µg/mL can eliminate 10^6^ CFU/mL of *C. difficile* within 30 min [134] and is comparable in efficacy to metronidazole and vancomycin in a model faecal environment. Less appealing, is the observation that lacticin has a similar effect on the resident gut microbiota as metronidazole and vancomycin, with a reduction in members of Firmicutes and Bacteroidetes and an increase in Proteobacteria [128]. This also adds to previous evidence that lacticin has a negative impact on lactobacilli and bifidobacteria [134].

Bioengineering of bacteriocins to make them more active against a particular pathogen offers potential to broaden the number of available therapeutics. Gebhart et al., discovered a novel type of bacteriocin, termed “diffocins”, which are produced by, and can kill *C. difficile* [135]. Diffocins are phage tail-like particles with bactericidal activity, similar to R-type pyocins produced by *Pseudomonas aeruginosa*. In a follow-up study, diffocins were modified to target common ribotype 027 strains and improve their physiochemical properties (e.g., pH and temperature stability). Highly potent and with an exquisitely narrow-spectrum of activity, these molecules could prevent colonisation by *C. difficile* following successful passage through the murine GI tract. In addition, treatment with the modified diffocins did not alter the commensal gut microbiota [136]. Taken together, these results suggest diffocins should be investigated further for potential use in humans. Furthermore, other bacteriocins, such as nisin, actagardine and mutacin have also been bioengineered to enhance their activity against *C. difficile* [137,138,139,140].

An overview of the preceding sections and their application to CDI are presented in Figure 1B,C.

## 10. Conclusions

*C. difficile* is a significant cause of morbidity and mortality worldwide. The emergence of hyper-virulent epidemic strains and increases in community-acquired CDI are worrying developments. While inherently resistant to many antibiotics, *C. difficile* has largely remained sensitive to first line antibiotic treatment options of metronidazole, vancomycin and fidaxomicin. The unique nature of CDI, where antibiotic therapy is actually a major risk factor for development of infection, has driven research to identify non-antibiotic alternatives. Such research has evolved in parallel with the explosion in microbiome research. Insights gained from the human microbiome, and more specifically the human gut microbiome, has revealed novel therapeutic options for CDI. In this review, we have outlined a number of such options. At this time, FMT appears to be best placed as an alternative to antibiotics, although adoption of FMT as a first-line treatment is not yet recommended. Future developments to identify what mix of strains best protect against *C. difficile* colonisation could alleviate some of the safety concerns associated with FMT. More refined therapies, such as spore treatments, have shown some promising initial results, but a recent failed clinical trial has dampened enthusiasm for this approach somewhat and further research is needed. Integrative approaches such as MWAS are still at an early stage, but have to the potential to identify important ecological interactions, metabolites and the dynamics of colonisation and progression of infection. WGS has shown promise in outbreak detection and identifying transmission events. Improvements to this technology could revolutionise pathogen diagnostics and infection control policies. The use of probiotics in preventing AAD and CDI remains somewhat controversial, with many conflicting studies on their efficacy. Much larger, randomised, double-blind, placebo controlled trials are needed in the future to clarify the potential positive benefits of probiotics. Microbial small molecule metabolites and antimicrobial compounds active against many pathogenic bacteria, including *C. difficile*, have been identified. The most difficult step is translating these discoveries into therapeutics efficacious and safe for use in humans. Overall, it is encouraging that many alternative and diverse therapeutics for *C. difficile* are under development. This review has not covered many other options, such as alternative antibiotics, synthetic drugs and vaccine-based approaches (reviewed in [141,142]). Future research and collaboration will undoubtedly identify further treatment options and therapeutic targets, while also progressing current options to a stage where they can make a meaningful contribution to treatment of CDI.

## References

[1] Qin J., Li R., Raes J., Arumugam M., Burgdorf K.S., Manichanh C., Nielsen T., Pons N., Levenez F., Yamada T. (2010). A human gut microbial gene catalogue established by metagenomic sequencing. Nature.

[2] Sender R., Fuchs S., Milo R. (2016). Are we really vastly outnumbered? Revisiting the ratio of bacterial to host cells in humans. Cell.

[3] Backhed F., Ding H., Wang T., Hooper L.V., Koh G.Y., Nagy A., Semenkovich C.F., Gordon J.I. (2004). The gut microbiota as an environmental factor that regulates fat storage. Proc. Natl. Acad. Sci. USA.

[4] Backhed F., Ley R.E., Sonnenburg J.L., Peterson D.A., Gordon J.I. (2005). Host-bacterial mutualism in the human intestine. Science.

[5] Hooper L.V., Gordon J.I. (2001). Commensal host-bacterial relationships in the gut. Science.

[6] Kurokawa K., Itoh T., Kuwahara T., Oshima K., Toh H., Toyoda A., Takami H., Morita H., Sharma V.K., Srivastava T.P. (2007). Comparative metagenomics revealed commonly enriched gene sets in human gut microbiomes. DNA Res..

[7] Rakoff-Nahoum S., Paglino J., Eslami-Varzaneh F., Edberg S., Medzhitov R. (2004). Recognition of commensal microflora by toll-like receptors is required for intestinal homeostasis. Cell.

[8] Samuel B.S., Gordon J.I. (2006). A humanized gnotobiotic mouse model of host-archaeal-bacterial mutualism. Proc. Natl. Acad. Sci. USA.

[9] Frank D.N., St Amand A.L., Feldman R.A., Boedeker E.C., Harpaz N., Pace N.R. (2007). Molecular-phylogenetic characterization of microbial community imbalances in human inflammatory bowel diseases. Proc. Natl. Acad. Sci. USA.

[10] Kostic A.D., Gevers D., Pedamallu C.S., Michaud M., Duke F., Earl A.M., Ojesina A.I., Jung J., Bass A.J., Tabernero J. (2012). Genomic analysis identifies association of fusobacterium with colorectal carcinoma. Genome Res..

[11] Turnbaugh P.J., Ley R.E., Mahowald M.A., Magrini V., Mardis E.R., Gordon J.I. (2006). An obesity-associated gut microbiome with increased capacity for energy harvest. Nature.

[12] Scott K.P., Antoine J.M., Midtvedt T., van Hemert S. (2015). Manipulating the gut microbiota to maintain health and treat disease. Microb. Ecol. Health Dis..

[13] Ananthakrishnan A.N. (2011). *Clostridium difficile* infection: Epidemiology, risk factors and management. Nat. Rev. Gastroenterol. Hepatol..

[14] Di Bella S., Ascenzi P., Siarakas S., Petrosillo N., di Masi A. (2016). *Clostridium difficile* toxins A and B: Insights into pathogenic properties and extraintestinal effects. Toxins.

[15] Just I., Wilm M., Selzer J., Rex G., von Eichel-Streiber C., Mann M., Aktories K. (1995). The enterotoxin from *Clostridium difficile* (toxa) monoglucosylates the rho proteins. J. Biol. Chem..

[16] Lyerly D.M., Krivan H.C., Wilkins T.D. (1988). *Clostridium difficile*: Its disease and toxins. Clin. Microbiol. Rev..

[17] Lyras D., O’Connor J.R., Howarth P.M., Sambol S.P., Carter G.P., Phumoonna T., Poon R., Adams V., Vedantam G., Johnson S. (2009). Toxin B is essential for virulence of *Clostridium difficile*. Nature.

[18] Kuehne S.A., Cartman S.T., Heap J.T., Kelly M.L., Cockayne A., Minton N.P. (2010). The role of toxin A and toxin B in *Clostridium difficile* infection. Nature.

[19] Kuehne S.A., Cartman S.T., Minton N.P. (2011). Both, toxin A and toxin B, are important in *Clostridium difficile* infection. Gut Microbes.

[20] Elliott B., Squire M.M., Thean S., Chang B.J., Brazier J.S., Rupnik M., Riley T.V. (2011). New types of toxin A-negative, toxin B-positive strains among clinical isolates of *Clostridium difficile* in Australia. J. Med. Microbiol..

[21] Janezic S., Marin M., Martin A., Rupnik M. (2015). A new type of toxin A-negative, toxin B-positive *Clostridium difficile* strain lacking a complete tcdA gene. J. Clin. Microbiol..

[22] Brouwer M.S., Roberts A.P., Hussain H., Williams R.J., Allan E., Mullany P. (2013). Horizontal gene transfer converts non-toxigenic *Clostridium difficile* strains into toxin producers. Nat. Commun..

[23] Crobach M.J., Dekkers O.M., Wilcox M.H., Kuijper E.J. (2009). European society of clinical microbiology and infectious diseases (ESCMID): Data review and recommendations for diagnosing *Clostridium difficile*-infection (CDI). Clin. Microbiol. Infect..

[24] Crogan N.L., Evans B.C. (2007). *Clostridium difficile*: An emerging epidemic in nursing homes. Geriatr. Nurs..

[25] Centers for Disease Control and Prevention (CDC) (2005). Severe *Clostridium difficile*-associated disease in populations previously at low risk—Four states, 2005. MMWR Morb. Mortal. Wkly. Rep..

[26] Cecil J.A. (2012). *Clostridium difficile*: Changing epidemiology, treatment and infection prevention measures. Curr. Infect. Dis. Rep..

[27] Kutty P.K., Woods C.W., Sena A.C., Benoit S.R., Naggie S., Frederick J., Evans S., Engel J., McDonald L.C. (2010). Risk factors for and estimated incidence of community-associated *Clostridium difficile* infection, North Carolina, USA. Emerg. Infect. Dis..

[28] Ghantoji S.S., Sail K., Lairson D.R., DuPont H.L., Garey K.W. (2010). Economic healthcare costs of *Clostridium difficile* infection: A systematic review. J. Hosp. Infect..

[29] McGlone S.M., Bailey R.R., Zimmer S.M., Popovich M.J., Tian Y., Ufberg P., Muder R.R., Lee B.Y. (2012). The economic burden of *Clostridium difficile*. Clin. Microbiol. Infect..

[30] Wilcox M.H., Cunniffe J.G., Trundle C., Redpath C. (1996). Financial burden of hospital-acquired *Clostridium difficile* infection. J. Hosp. Infect..

[31] Kuijper E.J., Coignard B., Tull P. (2006). Emergence of *Clostridium difficile*-associated disease in North America and Europe. Clin. Microbiol. Infect..

[32] Rupnik M., Wilcox M.H., Gerding D.N. (2009). *Clostridium difficile* infection: New developments in epidemiology and pathogenesis. Nat. Rev. Microbiol..

[33] Aldeyab M.A., Kearney M.P., Scott M.G., Aldiab M.A., Alahmadi Y.M., Darwish Elhajji F.W., Magee F.A., McElnay J.C. (2012). An evaluation of the impact of antibiotic stewardship on reducing the use of high-risk antibiotics and its effect on the incidence of *Clostridium difficile* infection in hospital settings. J. Antimicrob. Chemother..

[34] Hensgens M.P., Goorhuis A., Dekkers O.M., Kuijper E.J. (2012). Time interval of increased risk for *Clostridium difficile* infection after exposure to antibiotics. J. Antimicrob. Chemother..

[35] Talpaert M.J., Gopal Rao G., Cooper B.S., Wade P. (2011). Impact of guidelines and enhanced antibiotic stewardship on reducing broad-spectrum antibiotic usage and its effect on incidence of *Clostridium difficile* infection. J. Antimicrob. Chemother..

[36] Cohen S.H., Gerding D.N., Johnson S., Kelly C.P., Loo V.G., McDonald L.C., Pepin J., Wilcox M.H. (2010). Clinical practice guidelines for *Clostridium difficile* infection in adults: 2010 update by the society for healthcare epidemiology of America (SHEA) and the infectious diseases society of America (IDSA). Infect. Control Hosp. Epidemiol..

[37] McFarland L.V. (2005). Alternative treatments for *Clostridium difficile* disease: What really works?. J. Med. Microbiol..

[38] Kuijper E.J., Coignard B., Brazier J.S., Suetens C., Drudy D., Wiuff C., Pituch H., Reichert P., Schneider F., Widmer A.F. (2007). Update of *Clostridium difficile*-associated disease due to PCR ribotype 027 in Europe. Euro Surveill..

[39] Surawicz C.M., Brandt L.J., Binion D.G., Ananthakrishnan A.N., Curry S.R., Gilligan P.H., McFarland L.V., Mellow M., Zuckerbraun B.S. (2013). Guidelines for diagnosis, treatment, and prevention of *Clostridium difficile* infections. Am. J. Gastroenterol..

[40] Van Nood E., Vrieze A., Nieuwdorp M., Fuentes S., Zoetendal E.G., de Vos W.M., Visser C.E., Kuijper E.J., Bartelsman J.F., Tijssen J.G. (2013). Duodenal infusion of donor feces for recurrent *Clostridium difficile*. N. Engl. J. Med..

[41] Cammarota G., Masucci L., Ianiro G., Bibbo S., Dinoi G., Costamagna G., Sanguinetti M., Gasbarrini A. (2015). Randomised clinical trial: Faecal microbiota transplantation by colonoscopy vs. Vancomycin for the treatment of recurrent *Clostridium difficile* infection. Aliment. Pharmacol. Ther..

[42] Gough E., Shaikh H., Manges A.R. (2011). Systematic review of intestinal microbiota transplantation (fecal bacteriotherapy) for recurrent *Clostridium difficile* infection. Clin. Infect. Dis..

[43] Li Y.T., Cai H.F., Wang Z.H., Xu J., Fang J.Y. (2016). Systematic review with meta-analysis: Long-term outcomes of faecal microbiota transplantation for *Clostridium difficile* infection. Aliment. Pharmacol. Ther..

[44] Brandt L.J., Aroniadis O.C., Mellow M., Kanatzar A., Kelly C., Park T., Stollman N., Rohlke F., Surawicz C. (2012). Long-term follow-up of colonoscopic fecal microbiota transplant for recurrent *Clostridium difficile* infection. Am. J. Gastroenterol..

[45] Mattila E., Uusitalo-Seppala R., Wuorela M., Lehtola L., Nurmi H., Ristikankare M., Moilanen V., Salminen K., Seppala M., Mattila P.S. (2012). Fecal transplantation, through colonoscopy, is effective therapy for recurrent *Clostridium difficile* infection. Gastroenterology.

[46] Agrawal M., Aroniadis O.C., Brandt L.J., Kelly C., Freeman S., Surawicz C., Broussard E., Stollman N., Giovanelli A., Smith B. (2016). The long-term efficacy and safety of fecal microbiota transplant for recurrent, severe, and complicated *Clostridium difficile* infection in 146 elderly individuals. J. Clin. Gastroenterol..

[47] Baxter M., Colville A. (2016). Adverse events in faecal microbiota transplant: A review of the literature. J. Hosp. Infect..

[48] Bakken J.S., Borody T., Brandt L.J., Brill J.V., Demarco D.C., Franzos M.A., Kelly C., Khoruts A., Louie T., Martinelli L.P. (2011). Treating *Clostridium difficile* infection with fecal microbiota transplantation. Clin. Gastroenterol. Hepatol..

[49] Bojanova D.P., Bordenstein S.R. (2016). Fecal transplants: What is being transferred?. PLoS Biol..

[50] Sachs R., Edelstein C. (2015). Ensuring the safe and effective fda regulation of fecal microbiota transplantation. J. Law Biosci..

[51] Smith M.B., Kelly C., Alm E.J. (2014). Policy: How to regulate faecal transplants. Nature.

[52] Khanna S., Pardi D.S., Kelly C.R., Kraft C.S., Dhere T., Henn M.R., Lombardo M.J., Vulic M., Ohsumi T., Winkler J. (2016). A novel microbiome therapeutic increases gut microbial diversity and prevents recurrent *Clostridium difficile* infection. J. Infect. Dis..

[53] Gerding D.N., Meyer T., Lee C., Cohen S.H., Murthy U.K., Poirier A., Van Schooneveld T.C., Pardi D.S., Ramos A., Barron M.A. (2015). Administration of spores of nontoxigenic *Clostridium difficile* strain M3 for prevention of recurrent *C difficile* infection: A randomized clinical trial. JAMA J. Am. Med. Assoc..

[54] Dingle K.E., Griffiths D., Didelot X., Evans J., Vaughan A., Kachrimanidou M., Stoesser N., Jolley K.A., Golubchik T., Harding R.M. (2011). Clinical *Clostridium difficile*: Clonality and pathogenicity locus diversity. PLoS ONE.

[55] Chowdhury R.P., DeMaere M., Chapman T., Worden P., Charles I.G., Darling A.E., Djordjevic S.P. (2016). Comparative genomic analysis of toxin-negative strains of *Clostridium difficile* from humans and animals with symptoms of gastrointestinal disease. BMC Microbiol..

[56] Buffie C.G., Bucci V., Stein R.R., McKenney P.T., Ling L., Gobourne A., No D., Liu H., Kinnebrew M., Viale A. (2015). Precision microbiome reconstitution restores bile acid mediated resistance to *Clostridium difficile*. Nature.

[57] Petrof E.O., Gloor G.B., Vanner S.J., Weese S.J., Carter D., Daigneault M.C., Brown E.M., Schroeter K., Allen-Vercoe E. (2013). Stool substitute transplant therapy for the eradication of *Clostridium difficile* infection: ‘RePOOPulating’ the gut. Microbiome.

[58] Lawley T.D., Clare S., Walker A.W., Stares M.D., Connor T.R., Raisen C., Goulding D., Rad R., Schreiber F., Brandt C. (2012). Targeted restoration of the intestinal microbiota with a simple, defined bacteriotherapy resolves relapsing *Clostridium difficile* disease in mice. PLoS Pathog..

[59] Gilbert J.A., Quinn R.A., Debelius J., Xu Z.Z., Morton J., Garg N., Jansson J.K., Dorrestein P.C., Knight R. (2016). Microbiome-wide association studies link dynamic microbial consortia to disease. Nature.

[60] Koenigsknecht M.J., Theriot C.M., Bergin I.L., Schumacher C.A., Schloss P.D., Young V.B. (2015). Dynamics and establishment of *Clostridium difficile* infection in the murine gastrointestinal tract. Infect. Immun..

[61] Allegretti J.R., Kearney S., Li N., Bogart E., Bullock K., Gerber G.K., Bry L., Clish C.B., Alm E., Korzenik J.R. (2016). Recurrent *Clostridium difficile* infection associates with distinct bile acid and microbiome profiles. Aliment. Pharmacol. Ther..

[62] Ashton P.M., Peters T., Ameh L., McAleer R., Petrie S., Nair S., Muscat I., de Pinna E., Dallman T. (2015). Whole genome sequencing for the retrospective investigation of an outbreak of salmonella typhimurium DT 8. PLoS Curr..

[63] Jackson B.R., Tarr C., Strain E., Jackson K.A., Conrad A., Carleton H., Katz L.S., Stroika S., Gould L.H., Mody R.K. (2016). Implementation of nationwide real-time whole-genome sequencing to enhance listeriosis outbreak detection and investigation. Clin. Infect. Dis..

[64] Dominguez S.R., Anderson L.J., Kotter C.V., Littlehorn C.A., Arms L.E., Dowell E., Todd J.K., Frank D.N. (2015). Comparison of whole-genome sequencing and molecular-epidemiological techniques for *Clostridium difficile* strain typing. J. Pediatr. Infect. Dis. Soc..

[65] Harris S.R., Cartwright E.J., Torok M.E., Holden M.T., Brown N.M., Ogilvy-Stuart A.L., Ellington M.J., Quail M.A., Bentley S.D., Parkhill J. (2013). Whole-genome sequencing for analysis of an outbreak of meticillin-resistant staphylococcus aureus: A descriptive study. Lancet Infect. Dis..

[66] Lewis T., Loman N.J., Bingle L., Jumaa P., Weinstock G.M., Mortiboy D., Pallen M.J. (2010). High-throughput whole-genome sequencing to dissect the epidemiology of *Acinetobacter baumannii* isolates from a hospital outbreak. J. Hosp. Infect..

[67] Quick J., Cumley N., Wearn C.M., Niebel M., Constantinidou C., Thomas C.M., Pallen M.J., Moiemen N.S., Bamford A., Oppenheim B. (2014). Seeking the source of *Pseudomonas aeruginosa* infections in a recently opened hospital: An observational study using whole-genome sequencing. BMJ Open.

[68] Snitkin E.S., Zelazny A.M., Thomas P.J., Stock F., Henderson D.K., Palmore T.N., Segre J.A. (2012). Tracking a hospital outbreak of carbapenem-resistant *Klebsiella pneumoniae* with whole-genome sequencing. Sci. Transl. Med..

[69] Kumar N., Miyajima F., He M., Roberts P., Swale A., Ellison L., Pickard D., Smith G., Molyneux R., Dougan G. (2016). Genome-based infection tracking reveals dynamics of *Clostridium difficile* transmission and disease recurrence. Clin. Infect. Dis..

[70] Mac Aogain M., Moloney G., Kilkenny S., Kelleher M., Kelleghan M., Boyle B., Rogers T.R. (2015). Whole-genome sequencing improves discrimination of relapse from reinfection and identifies transmission events among patients with recurrent *Clostridium difficile* infections. J. Hosp. Infect..

[71] Jia H., Du P., Yang H., Zhang Y., Wang J., Zhang W., Han G., Han N., Yao Z., Wang H. (2016). Nosocomial transmission of *Clostridium difficile* ribotype 027 in a chinese hospital, 2012–2014, traced by whole genome sequencing. BMC Genom..

[72] Eyre D.W., Tracey L., Elliott B., Slimings C., Huntington P.G., Stuart R.L., Korman T.M., Kotsiou G., McCann R., Griffiths D. (2015). Emergence and spread of predominantly community-onset *Clostridium difficile* PCR ribotype 244 infection in Australia, 2010 to 2012. Euro Surveill..

[73] Steglich M., Nitsche A., von Muller L., Herrmann M., Kohl T.A., Niemann S., Nubel U. (2015). Tracing the spread of *Clostridium difficile* ribotype 027 in germany based on bacterial genome sequences. PLoS ONE.

[74] Knetsch C.W., Connor T.R., Mutreja A., van Dorp S.M., Sanders I.M., Browne H.P., Harris D., Lipman L., Keessen E.C., Corver J. (2014). Whole genome sequencing reveals potential spread of *Clostridium difficile* between humans and farm animals in the netherlands, 2002 to 2011. Euro Surveill..

[75] Quick J., Ashton P., Calus S., Chatt C., Gossain S., Hawker J., Nair S., Neal K., Nye K., Peters T. (2015). Rapid draft sequencing and real-time nanopore sequencing in a hospital outbreak of salmonella. Genome Biol..

[76] Quick J., Loman N.J., Duraffour S., Simpson J.T., Severi E., Cowley L., Bore J.A., Koundouno R., Dudas G., Mikhail A. (2016). Real-time, portable genome sequencing for ebola surveillance. Nature.

[77] Loose M., Malla S., Stout M. (2016). Real-time selective sequencing using nanopore technology. Nat. Methods.

[78] Kwong J.C., McCallum N., Sintchenko V., Howden B.P. (2015). Whole genome sequencing in clinical and public health microbiology. Pathology.

[79] Allard M.W. (2016). The future of whole-genome sequencing for public health and the clinic. J. Clin. Microbiol..

[80] Fricke W.F., Rasko D.A. (2014). Bacterial genome sequencing in the clinic: Bioinformatic challenges and solutions. Nat. Rev. Genet..

[81] Mellmann A., Bletz S., Boking T., Kipp F., Becker K., Schultes A., Prior K., Harmsen D. (2016). Real-time genome sequencing of resistant bacteria provides precision infection control in an institutional setting. J. Clin. Microbiol..

[82] Hill C., Guarner F., Reid G., Gibson G.R., Merenstein D.J., Pot B., Morelli L., Canani R.B., Flint H.J., Salminen S. (2014). Expert consensus document. The international scientific association for probiotics and prebiotics consensus statement on the scope and appropriate use of the term probiotic. Nat. Rev. Gastroenterol. Hepatol..

[83] Culligan E.P., Hill C., Sleator R.D. (2009). Probiotics and gastrointestinal disease: Successes, problems and future prospects. Gut Pathog..

[84] Sleator R.D. (2010). Probiotic therapy—Recruiting old friends to fight new foes. Gut Pathog..

[85] Maziade P.J., Pereira P., Goldstein E.J. (2015). A decade of experience in primary prevention of *Clostridium difficile* infection at a community hospital using the probiotic combination *Lactobacillus acidophilus* CL1285, *Lactobacillus casei* LBC80R, and *Lactobacillus rhamnosus* CLR2 (Bio-K+). Clin. Infect. Dis..

[86] Gao X.W., Mubasher M., Fang C.Y., Reifer C., Miller L.E. (2010). Dose-response efficacy of a proprietary probiotic formula of *Lactobacillus acidophilus* CL1285 and *Lactobacillus casei* LBC80R for antibiotic-associated diarrhea and *Clostridium difficile*-associated diarrhea prophylaxis in adult patients. Am. J. Gastroenterol..

[87] Goldenberg J.Z., Ma S.S., Saxton J.D., Martzen M.R., Vandvik P.O., Thorlund K., Guyatt G.H., Johnston B.C. (2013). Probiotics for the prevention of *Clostridium difficile*-associated diarrhea in adults and children. Cochrane Database Syst. Rev..

[88] Johnson S., Maziade P.J., McFarland L.V., Trick W., Donskey C., Currie B., Low D.E., Goldstein E.J. (2012). Is primary prevention of *Clostridium difficile* infection possible with specific probiotics?. Int. J. Infect. Dis..

[89] Johnston B.C., Ma S.S., Goldenberg J.Z., Thorlund K., Vandvik P.O., Loeb M., Guyatt G.H. (2012). Probiotics for the prevention of *Clostridium difficile*-associated diarrhea: A systematic review and meta-analysis. Ann. Int. Med..

[90] McFarland L.V., Surawicz C.M., Greenberg R.N., Fekety R., Elmer G.W., Moyer K.A., Melcher S.A., Bowen K.E., Cox J.L., Noorani Z. (1994). A randomized placebo-controlled trial of *Saccharomyces boulardii* in combination with standard antibiotics for *Clostridium difficile* disease. J. Am. Med.Assoc..

[91] Surawicz C.M., McFarland L.V., Greenberg R.N., Rubin M., Fekety R., Mulligan M.E., Garcia R.J., Brandmarker S., Bowen K., Borjal D. (2000). The search for a better treatment for recurrent *Clostridium difficile* disease: Use of high-dose vancomycin combined with *Saccharomyces boulardii*. Clin. Infect. Dis..

[92] Pozzoni P., Riva A., Bellatorre A.G., Amigoni M., Redaelli E., Ronchetti A., Stefani M., Tironi R., Molteni E.E., Conte D. (2012). *Saccharomyces boulardii* for the prevention of antibiotic-associated diarrhea in adult hospitalized patients: A single-center, randomized, double-blind, placebo-controlled trial. Am. J. Gastroenterol..

[93] Pochapin M. (2000). The effect of probiotics on *Clostridium difficile* diarrhea. Am. J. Gastroenterol..

[94] Wullt M., Hagslatt M.L., Odenholt I. (2003). Lactobacillus plantarum 299v for the treatment of recurrent *Clostridium difficile*-associated diarrhoea: A double-blind, placebo-controlled trial. Scand. J. Infect. Dis..

[95] Allen S.J., Wareham K., Wang D., Bradley C., Hutchings H., Harris W., Dhar A., Brown H., Foden A., Gravenor M.B. (2013). Lactobacilli and bifidobacteria in the prevention of antibiotic-associated diarrhoea and *Clostridium difficile* diarrhoea in older inpatients (PLACIDE): A randomised, double-blind, placebo-controlled, multicentre trial. Lancet.

[96] Allen S.J., Wareham K., Wang D., Bradley C., Sewell B., Hutchings H., Harris W., Dhar A., Brown H., Foden A. (2013). A high-dose preparation of lactobacilli and bifidobacteria in the prevention of antibiotic-associated and *Clostridium difficile* diarrhoea in older people admitted to hospital: A multicentre, randomised, double-blind, placebo-controlled, parallel arm trial (placide). Health Technol. Assess..

[97] Evans C.T., Johnson S. (2015). Prevention of *Clostridium difficile* infection with probiotics. Clin. Infect. Dis..

[98] Sleator R.D. (2012). Digital biology: A new era has begun. Bioengineered.

[99] Sleator R.D. (2014). The synthetic biology future. Bioengineered.

[100] Sheehan V.M., Sleator R.D., Fitzgerald G.F., Hill C. (2006). Heterologous expression of Betl, a betaine uptake system, enhances the stress tolerance of Lactobacillus salivarius UCC118. Appl. Environ. Microbiol..

[101] Sheehan V.M., Sleator R.D., Hill C., Fitzgerald G.F. (2007). Improving gastric transit, gastrointestinal persistence and therapeutic efficacy of the probiotic strain Bifidobacterium breve UCC2003. Microbiology.

[102] Sleator R.D. (2015). Designer probiotics: Development and applications in gastrointestinal health. World J. Gastrointest. Pathophysiol..

[103] Sleator R.D., Hill C. (2006). Patho-biotechnology: Using bad bugs to do good things. Curr. Opin. Biotechnol..

[104] Sleator R.D., Hill C. (2007). Patho-biotechnology; using bad bugs to make good bugs better. Sci. Prog..

[105] Watson D., Sleator R.D., Hill C., Gahan C.G. (2008). Enhancing bile tolerance improves survival and persistence of Bifidobacterium and Lactococcus in the murine gastrointestinal tract. BMC Microbiol..

[106] Hwang I.Y., Tan M.H., Koh E., Ho C.L., Poh C.L., Chang M.W. (2014). Reprogramming microbes to be pathogen-seeking killers. ACS Synth. Biol..

[107] Saeidi N., Wong C., Lo T., Nguyen H., Ling H., Leong S., Poh C., Chang M. (2011). Engineering microbes to sense and eradicate *Pseudomonas aeruginosa*, a human pathogen. Mol. Syst. Biol..

[108] Paton A.W., Morona R., Paton J.C. (2000). A new biological agent for treatment of shiga toxigenic *Escherichia coli* infections and dysentery in humans. Nat. Med..

[109] Focareta A., Paton J.C., Morona R., Cook J., Paton A.W. (2006). A recombinant probiotic for treatment and prevention of cholera. Gastroenterology.

[110] Paton A.W., Jennings M.P., Morona R., Wang H., Focareta A., Roddam L.F., Paton J.C. (2005). Recombinant probiotics for treatment and prevention of enterotoxigenic *Escherichia coli* diarrhea. Gastroenterology.

[111] Din M.O., Danino T., Prindle A., Skalak M., Selimkhanov J., Allen K., Julio E., Atolia E., Tsimring L.S., Bhatia S.N. (2016). Synchronized cycles of bacterial lysis forin vivo delivery. Nature.

[112] Sleator R.D., Hill C. (2008). Designer probiotics: A potential therapeutic for *Clostridium difficile*?. J. Med. Microbiol..

[113] Antunes L.C., Han J., Ferreira R.B., Lolic P., Borchers C.H., Finlay B.B. (2011). Effect of antibiotic treatment on the intestinal metabolome. Antimicrob. Agents Chemother..

[114] Antunes L.C., McDonald J.A., Schroeter K., Carlucci C., Ferreira R.B., Wang M., Yurist-Doutsch S., Hira G., Jacobson K., Davies J. (2014). Antivirulence activity of the human gut metabolome. mBio.

[115] Darkoh C., DuPont H.L., Norris S.J., Kaplan H.B. (2015). Toxin synthesis by *Clostridium difficile* is regulated through quorum signaling. mBio.

[116] Bender K.O., Garland M., Ferreyra J.A., Hryckowian A.J., Child M.A., Puri A.W., Solow-Cordero D.E., Higginbottom S.K., Segal E., Banaei N. (2015). A small-molecule antivirulence agent for treating *Clostridium difficile* infection. Sci. Transl. Med..

[117] Yamaguchi T., Sano K., Takakura K., Saito I., Shinohara Y., Asano T., Yasuhara H. (1998). Ebselen in acute ischemic stroke: A placebo-controlled, double-blind clinical trial. Ebselen study group. Stroke J. Cereb. Circ..

[118] Tam J., Beilhartz G.L., Auger A., Gupta P., Therien A.G., Melnyk R.A. (2015). Small molecule inhibitors of *Clostridium difficile* toxin B-induced cellular damage. Chem. Biol..

[119] Ripert G., Racedo S.M., Elie A.M., Jacquot C., Bressollier P., Urdaci M.C. (2016). Secreted compounds of the probiotic bacillus clausii strain O/C inhibit the cytotoxic effects induced by *Clostridium difficile* and Bacillus cereus toxins. Antimicrob. Agents Chemother..

[120] Huelsenbeck J., Dreger S.C., Gerhard R., Fritz G., Just I., Genth H. (2007). Upregulation of the immediate early gene product rhob by exoenzyme C3 from clostridium limosum and toxin B from *Clostridium difficile*. Biochemistry.

[121] Cotter P.D., Ross R.P., Hill C. (2013). Bacteriocins—A viable alternative to antibiotics?. Nat. Rev. Microbiol..

[122] Donia M.S., Cimermancic P., Schulze C.J., Wieland Brown L.C., Martin J., Mitreva M., Clardy J., Linington R.G., Fischbach M.A. (2014). A systematic analysis of biosynthetic gene clusters in the human microbiome reveals a common family of antibiotics. Cell.

[123] Morton J.T., Freed S.D., Lee S.W., Friedberg I. (2015). A large scale prediction of bacteriocin gene blocks suggests a wide functional spectrum for bacteriocins. BMC Bioinform..

[124] Walsh C.J., Guinane C.M., Hill C., Ross R.P., O’Toole P.W., Cotter P.D. (2015). In silico identification of bacteriocin gene clusters in the gastrointestinal tract, based on the human microbiome project’s reference genome database. BMC Microbiol..

[125] Zipperer A., Konnerth M.C., Laux C., Berscheid A., Janek D., Weidenmaier C., Burian M., Schilling N.A., Slavetinsky C., Marschal M. (2016). Human commensals producing a novel antibiotic impair pathogen colonization. Nature.

[126] Kommineni S., Bretl D.J., Lam V., Chakraborty R., Hayward M., Simpson P., Cao Y., Bousounis P., Kristich C.J., Salzman N.H. (2015). Bacteriocin production augments niche competition by enterococci in the mammalian gastrointestinal tract. Nature.

[127] Rea M.C., Sit C.S., Clayton E., O’Connor P.M., Whittal R.M., Zheng J., Vederas J.C., Ross R.P., Hill C. (2010). Thuricin CD, a posttranslationally modified bacteriocin with a narrow spectrum of activity against *Clostridium difficile*. Proc. Natl. Acad. Sci. USA.

[128] Rea M.C., Dobson A., O’Sullivan O., Crispie F., Fouhy F., Cotter P.D., Shanahan F., Kiely B., Hill C., Ross R.P. (2011). Effect of broad- and narrow-spectrum antimicrobials on *Clostridium difficile* and microbial diversity in a model of the distal colon. Proc. Natl. Acad. Sci. USA.

[129] Rea M.C., Alemayehu D., Casey P.G., O’Connor P.M., Lawlor P.G., Walsh M., Shanahan F., Kiely B., Ross R.P., Hill C. (2014). Bioavailability of the anti-clostridial bacteriocin thuricin CD in gastrointestinal tract. Microbiology.

[130] Murphy K., O’Sullivan O., Rea M.C., Cotter P.D., Ross R.P., Hill C. (2011). Genome mining for radical sam protein determinants reveals multiple sactibiotic-like gene clusters. PLoS ONE.

[131] Bartoloni A., Mantella A., Goldstein B.P., Dei R., Benedetti M., Sbaragli S., Paradisi F. (2004). In vitro activity of nisin against clinical isolates of *Clostridium difficile*. J. Chemother..

[132] Le Lay C., Fernandez B., Hammami R., Ouellette M., Fliss I. (2015). On lactococcus lactis ul719 competitivity and nisin (Nisaplin^®^) capacity to inhibit *Clostridium difficile* in a model of human colon. Front. Microbiol..

[133] Ryan M.P., Rea M.C., Hill C., Ross R.P. (1996). An application in cheddar cheese manufacture for a strain of Lactococcus lactis producing a novel broad-spectrum bacteriocin, lacticin 3147. Appl. Environ. Microbiol..

[134] Rea M.C., Clayton E., O’Connor P.M., Shanahan F., Kiely B., Ross R.P., Hill C. (2007). Antimicrobial activity of lacticin 3147 against clinical *Clostridium difficile* strains. J. Med. Microbiol..

[135] Gebhart D., Williams S.R., Bishop-Lilly K.A., Govoni G.R., Willner K.M., Butani A., Sozhamannan S., Martin D., Fortier L.C., Scholl D. (2012). Novel high-molecular-weight, R-type bacteriocins of *Clostridium difficile*. J. Bacteriol..

[136] Gebhart D., Lok S., Clare S., Tomas M., Stares M., Scholl D., Donskey C.J., Lawley T.D., Govoni G.R. (2015). A modified R-type bacteriocin specifically targeting *Clostridium difficile* prevents colonization of mice without affecting gut microbiota diversity. mBio.

[137] Boakes S., Ayala T., Herman M., Appleyard A.N., Dawson M.J., Cortes J. (2012). Generation of an actagardine a variant library through saturation mutagenesis. Appl. Microbiol. Biotechnol..

[138] Chen S., Wilson-Stanford S., Cromwell W., Hillman J.D., Guerrero A., Allen C.A., Sorg J.A., Smith L. (2013). Site-directed mutations in the lanthipeptide mutacin 1140. Appl. Environ. Microbiol..

[139] Crowther G.S., Baines S.D., Todhunter S.L., Freeman J., Chilton C.H., Wilcox M.H. (2013). Evaluation of NVB302 versus vancomycin activity in an in vitro human gut model of *Clostridium difficile* infection. J. Antimicrob. Chemother..

[140] Field D., Quigley L., O’Connor P.M., Rea M.C., Daly K., Cotter P.D., Hill C., Ross R.P. (2010). Studies with bioengineered nisin peptides highlight the broad-spectrum potency of Nisin V. Microb. Biotechnol..

[141] Jarrad A.M., Karoli T., Blaskovich M.A., Lyras D., Cooper M.A. (2015). *Clostridium difficile* drug pipeline: Challenges in discovery and development of new agents. J. Med. Chem..

[142] Mathur H., Rea M.C., Cotter P.D., Ross R.P., Hill C. (2014). The potential for emerging therapeutic options for *Clostridium difficile* infection. Gut Microbes.

